# Co‐Creation of a Study Protocol to Assess the Effect of Transcranial Direct Current Stimulation in the Management of Fatigue in Children and Young People With Acquired Brain Injury (Fatiguebrain‐tDCS)

**DOI:** 10.1111/hex.70706

**Published:** 2026-06-14

**Authors:** Chandrasekar Rathinam, Gemma Heath, Rajat Gupta, Evangeline Wassmer, Emily McElroy, Helen Horn, Davinia Fernandez‐Espejo

**Affiliations:** ^1^ School of Psychology University of Birmingham Birmingham UK; ^2^ Centre for Human Brain Health University of Birmingham Birmingham UK; ^3^ Birmingham Women and Childrened Hospital, Steelhouse Lane Birmingham UK; ^4^ Aston Institute of Health & Neurodevelopment Aston University Birmingham UK; ^5^ Parents, Members of the Patient Public Involvement and Engagement group UK

**Keywords:** acquired brain injury, children and young people, co‐creation, intervention development, patient public involvement and engagement, stakeholders

## Abstract

**Background:**

Children and young people (CYP) with moderate/severe acquired brain injury (ABI) often have problems with chronic cognitive fatigue. Methylphenidate, a controlled drug, and cognitive behavioural therapy have been used to manage fatigue, but their effects are not fully established, and they are not widely available. Transcranial direct current stimulation (tDCS) is a non‐invasive brain stimulation technique that has shown promise for managing fatigue in adults with neurological conditions. However, no studies have used tDCS to manage fatigue in CYP with ABI nor have involved patients to co‐create the intervention or research protocol.

**Objective:**

To co‐create a trial protocol for a study examining the feasibility of using tDCS as an intervention to manage fatigue in CYP with ABI and provide proof‐of‐concept for its efficacy through patient public involvement and engagement (PPIE).

**Design:**

Our co‐creation approach encompassed three key stages: co‐define, co‐design, and co‐refine to actively bring stakeholders together. The co‐define phase consisted of discussing end‐users' experiences and needs related to fatigue. We then co‐designed an intervention protocol to address fatigue using tDCS. The final phase invited stakeholder feedback to co‐refine the tDCS intervention, ensuring it was acceptable and sustainable.

**Setting and Participants:**

Stakeholders (*n* = 42) included CYP with ABI and their parents as well as members of an established young persons' advisory group. Stakeholders participated in in‐person, online and hybrid focus groups, 1:1 meetings, and surveys, over five stages.

**Results:**

We followed the Medical Research Council guidelines for intervention development and used design thinking approaches to co‐create the tDCS intervention. This process led to the development of a study protocol where tDCS is delivered at home rather than in the hospital and to monitor sessions remotely via an online platform. The treatment duration was extended to 4 weeks to maximise the likelihood of detecting beneficial effects. Stakeholder contribution also led to the inclusion of children with neurodiversity and to making the research more accessible for everyone.

**Discussion and Conclusions:**

PPIE members were involved in co‐defining the problem, co‐designing the intervention, determining treatment parameters, selecting efficacy outcome measures, identifying barriers to adherence, developing mitigating strategies, and co‐producing and reviewing the research study protocol. This ongoing stakeholder involvement shaped the co‐creation process, balancing idealistic approaches with feasible steps. Our protocol considered implementation barriers from the start and balanced scientific needs with patient and family priorities. The PPIE members will continue to be involved in conducting the study and designing qualitative work, reinforcing their ongoing engagement.

**Patient or Public Contribution:**

To develop this study protocol, the PPIE members were actively involved in defining the problem, creating the intervention, determining treatment parameters, selecting the relevant outcome measures, identifying barriers to adherence, developing mitigating strategies, reviewing the research study protocol and writing the manuscript.

**Abbreviations**
ABI: Acquired Brain Injury
CBT: Cognitive Behavioural Therapy
CSN: Cortico–striatal network
CYP: Children and Young People
DLPFC: Dorsolateral prefrontal cortex
GRIPP2: Guidance for Reporting Involvement of Patients and the Public 2
MRI: Magnetic Resonance Image
NHS: National Health Services
NIBS: Non‐invasive Brain Stimulation
NIHR: National Institute for Health and Care Research
PPIE: Patient Public Involvement and Engagement
RCTs: Randomised Controlled Trials
tDCS: transcranial Direct Current Stimulation
YPAG: Young Persons' Advisory Group

## 1 Background

Children and young people (CYP) with moderate to severe acquired brain injury (ABI) often develop comorbidities during the sub‐acute and chronic phases [1]. Though clinical characteristics can vary for traumatic and non‐traumatic ABI, during the acute and sub‐acute stages, these patients typically present with muscle weakness, movement difficulties, poor sleep regulation, memory loss and challenges to emotional regulation [2]. After the subacute stage, a substantial number of CYP also develop further problems, including decreased attention and concentration, altered behaviour, processing delay, memory loss, motor problems, and physical and cognitive fatigue [3, 4, 5]. Chronic fatigue, whether physical, mental, or both, profoundly limits quality of life for CYP and their families, impacting learning and social activities [6, 7, 8].

While pharmacological (e.g., Methylphenidate) and non‐pharmacological (e.g., Cognitive Behavioural Therapy (CBT)) treatments have been used to manage fatigue, their limitations are well‐established. The UK National Institute for Health and Care Excellence guidelines on rehabilitation for chronic neurological disorders, including ABI, do not specify CBT as a standard or required therapy for children with ABI [9]. Paediatric neurorehabilitation services and access to specialist paediatric neuropsychology are also limited due to local National Health Services (NHS) commissioning priorities. CBT provision is not therefore, widely available for CYP with ABI, and its impact on fatigue in paediatric neurological conditions is not fully understood [10]. Similarly, Methylphenidate, a controlled drug, has an uncertain level of effectiveness for improving fatigue in CYP and is therefore seldom prescribed [11, 12].

In recent years, non‐invasive brain stimulation (NIBS) has emerged as a treatment option for a range of neurological conditions, including those that are resistant to medication [13, 14, 15]. To date, the most widely used NIBS methods are transcranial magnetic stimulation and transcranial direct current stimulation (tDCS). Both have been used to treat fatigue in adult neurological conditions and have shown improved functional outcomes [16, 17, 18, 19], with tDCS having the advantage of being more portable, affordable, and easy to use [20]. tDCS can modulate neural excitability in a polarity‐specific manner [21] causing changes in the neurotransmission system, synaptic microenvironment and neural connectivity [22] to produce therapeutic effects [23]. Magnetic resonance imaging (MRI) studies in paediatric neurological conditions have identified that disrupted cortico–striatal network (CSN) functional connectivity is related to cognitive fatigue [24]. This CSN dysfunction is associated with poor motivation, inefficient and costly processing, and cognitive and affective functioning deficits, which leads to both increased cognitive fatigue [25] as well as an effort‐reward imbalance in the subjective experience of such cognitive fatigue [24]. A key node in the CSN is the left dorsolateral prefrontal cortex (DLPFC), which is the most common target in tDCS interventions for fatigue in adults [26, 27], partially due to its surface position in the cortex, making it accessible to non‐invasive transcranial approaches.

Studies using tDCS to manage fatigue in adults use a classic montage with the anode over the left DLPFC (F3) and the cathode over the contralateral supraorbital area (near Fp2) [26, 27]. tDCS is considered safe to use in paediatric groups, with a recent expert review highlighting that the frequency, magnitude and type of adverse events is comparable to that across healthy adults, albeit with studies in children typically using lower intensities [28]. Crucially, the risk of tDCS‐induced seizures is extremely low [29], with no published cases to date in children [30]. This is critical in CYP with ABI, as the risk of epilepsy or seizure is high [31]. Supervised tDCS has been used at home to treat children [32] and adults with various neurological conditions [33], with good adherence and a good safety profile (i.e. no serious adverse events) [34].

A recent scoping review identified only nine studies using tDCS in CYP with ABI between 2006 and 2023 [35]. Seven were single case reports, one a quasi‐randomised controlled trial involving 12 participants, and one a retrospective study involving 44 children with mild traumatic brain injury. The tDCS frequency (3–21 sessions), duration (10–35 min per session), and length (3 days to 8 weeks) varied across studies. Findings suggested improvements in motor function, headaches, and sensory processing. However, none of these studies included fatigue as an outcome. To the authors' knowledge, there are no available randomised controlled trials (RCTs) testing tDCS for CYP with ABI to manage fatigue. A well‐controlled RCT is thus required to establish the effectiveness of tDCS to manage fatigue in children, the dosage and the number and length of sessions to obtain an effect need. In addition, it is important to document any potential barriers to accessing treatment and complying with protocol instructions.

Alongside scientific considerations, there is ample evidence to support the involvement of all stakeholders in the development of interventions and protocol studies. This is crucial for designing interventions and research designs that are likely to be acceptable to end users [36, 37]. The three ‘Co‐’ frameworks, which include co‐production, co‐creation, and co‐design, are methods that actively bring together stakeholders under the umbrella of ‘Participatory Action Research’ [38]. Co‐design involves integrating stakeholders' views and experiences when creating new interventions, bridging the gap between development and implementation [39]. The UK National Institute for Health and Care Research (NIHR) recognises the value of researchers, practitioners, patients, and the public collaborating to conduct studies, demonstrated in their requirement for active Patient and Public Involvement and Engagement (PPIE) in publicly funded research [40]. This approach also facilitates the involvement of underrepresented and minority group members [41], seeking to create more contextually appropriate solutions. Similarly, the Medical Research Council guidelines for designing complex interventions emphasise the importance of considering contextual factors and implementation issues from the outset [42]. In the absence of previous studies using tDCS to manage fatigue problems in CYP with ABI, developing a tDCS intervention and research protocol is considered complex.

The aim of this project was to work with families of CYP with ABI to co‐create a trial protocol for a study examining the feasibility of using tDCS for managing fatigue in CYP with ABI (FatigueBraIn‐tDCS). Working with families in this context was crucial for creating an intervention that would be considered feasible. A critical factor in post ABI fatigue is that chronic problems often require behavioural changes for the sustainable implementation of interventions [43, 44]. By involving parents and patients in key decisions regarding intervention design and implementation (e.g. treatment timing, duration, location etc), we would expect the resulting intervention to be acceptable and sustainable from the outset, increasing the likelihood of adherence within a trial and beyond. In this paper, we describe the collaborative, iterative and dialogue‐based processes undertaken to co‐design a complex neurological intervention (tDCS) and feasibility trial protocol, providing a worked example of applying co‐creation methodology. Guidance for the Reporting of Involvement of Patients and the Public in research (GRIPP2) is followed, to ensure transparency in our reporting [45] (Appendix 1).

## 2 Methods and Findings

**Figure 1:**
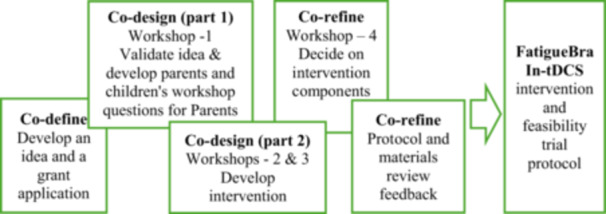
Intervention development process using an overlapping iterative co‐creation approach.

We adapted a co‐creation method reported by Pearce et al [46]. Co‐creation is an inclusive process that begins by co‐defining the problem with stakeholders and exploring potential solutions [47]. Co‐creation builds on PPIE by shifting the research paradigm from simply consulting with patients (involvement) to actively collaborating with them as equal partners [48], and the overarching co‐creation components are explained in Figure 1.

**Figure 2:**
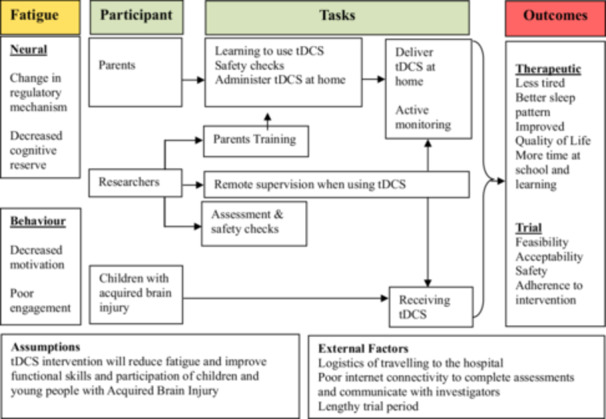
Logic model for FatigueBraIn‐tDCS.

The initial *Co‐define* phase consisted of discussing fatigue in CYP with ABI with parents and CYP to identify the experience and needs of end‐users. This validated the problem of fatigue for children with ABI and contributed to stage 2, *Co‐design* of a tDCS intervention and associated feasibility trial protocol. The final stage invited feedback from stakeholders to *Co‐refine* the intervention and protocol, ensuring they were acceptable and sustainable [47]. Methods and results of the iterative co‐creation processes are described in the following section, along with a logic model underpinning the proposed intervention (Figure 2).

### 2.1 Co‐Define

#### 2.1.1 Methods

To develop the focus and scope of the FatigueBraIn‐tDCS project (and application for funding), CYP with ABI and their parents were consulted via discussion in the neurorehabilitation outpatient clinic of a UK specialist children's hospital, followed up by a short survey. Families were informed about tDCS as a treatment option, and provided with a brief summary of evidence, mechanisms of action, and associated risks, and were asked to provide their views. We did not discuss alternative non‐invasive brain stimulation approaches. In the absence of medical management and CBT availability, parents were keen to explore tDCS as a possible treatment option.

Following funding approval, an in‐person workshop was arranged with a well‐established NIHR Young Persons' Advisory Group (NIHR‐YPAG) at a specialist children's hospital, to gather more in‐depth views from young people. The NIHR YPAG consists of ex‐patients, siblings, and interested young persons from the local community. They meet every month to help shape health research ideas related to CYP. NIHR‐YPAG group members who participated in this work consisted of eight participants (aged 10−16 years) and two YPAG coordinators, who attended a 1 hour session. Participants were paired into four groups to discuss issues related to the research, including gaps in existing evidence, appropriate terminology, barriers to recruitment, and eliciting ideas for engaging CYP with ABI in research. YPAG members presented their views, using ‘Post‐it’ notes to facilitate detailed responses for each issue. Verbal responses were audio‐recorded with participant permission for summary purposes. The YPAG group also advised on the construction of questions and prompts for use in a subsequent workshop involving parents and children with ABI, as well as commenting on the invitation to create a project‐specific stakeholder group. The NIHR funded travel expenses and honorarium for the YPAG members.

#### 2.1.2 Results

Informal discussion with families of children with ABI in the outpatient clinic, and five survey responses, all confirmed fatigue as a significant problem for CYP with ABI, and thus an important area for research. The NIHR YPAG group also considered our FatigueBraIn‐tDCS study idea to be important. They raised key questions about feasibility, including whether participants needed to use tDCS on a long‐term basis and what the impact of tDCS might be on other issues, such as concentration. Young people recognised that these were some of the uncertainties of the study but recommended discussing these topics openly and expanding on them with examples related to long‐term use (time to see changes in fatigue levels, maintenance dose, etc.) when approaching participants.

It was important for YPAG members that no participants should feel that something was wrong with them in terms of their ABI, and that plain and accessible language be used to explain processes. They suggested adapting communication styles to suit individual participants, suggesting this would help to make children and parents feel safe, comfortable and happy to try tDCS. The YPAG recognised children's variable levels of understanding. Subsequently, they recommended using pictures and animated videos, as well as utilising the experience of those who had received tDCS before. Young people recommended engaging an interpreter to manage language barriers and promote inclusivity.

Regarding treatment intensity, the YPAG suggested starting with 15 min and progressively increasing the time as tolerated. They also suggested using tDCS treatment in the morning to help with fatigue later in the day while at school. Although school and friends were identified as important, young people rejected treatment delivery at school for reasons of privacy. Instead, young people recommended that parents or clinicians support treatment delivery at home or in hospital.

YPAG members did not view electrical or current stimulation as unacceptable, but suggested that side effects, such as a burning sensation, may be of concern. Showing images of tDCS was thought to help visualise the treatment and alleviate misconceptions. They also recommended explanations of the treatment to be age‐appropriate, focusing on the long‐term benefits of reduced fatigue. Young people also thought parents required explanations of the benefits and risks and should be invited to help explain the treatment and side effects. The YPAG felt that the potential benefit of tDCS would encourage participants to try the treatment. They recommended relaxation strategies to support children when using tDCS (e.g. colouring, painting, meditating, watching TV, and whatever they enjoy).

### 2.2 Co‐Design Part 1

#### 2.2.1 Methods

The first co‐design workshop involved children with ABI and their parents. Recruitment was supported by The Stroke Charity, UK which advertised the stakeholder workshop on their social media and posted an advert to parents across the UK. Young people with ABI and parents who previously contributed to the funding application were also invited. Two online workshops were conducted, one with CYP and one with parents. Taking an online approach had the benefit of reduced participant burden and being able to include families who were geographically dispersed. It also enabled participants from different age groups, socio‐economic backgrounds, and language and cultural backgrounds to attend the meeting. Workshops were held sequentially in the early evening, CYP first, followed by parents. Workshops focused on discussing families' understanding of tDCS treatment, outcomes of importance, factors influencing potential participation and acceptable parameters.

The first workshop involved CYP with ABI and lasted for approximately 1 hour. Workshops were designed to be supportive, with parents encouraged to stay with their child to support communication. A short presentation of the research was provided, followed by an informal discussion loosely structured by a set of pre‐determined questions (e.g. What changes would you like to see after the treatment? What makes you consider or not consider this treatment?).

Parents attended a separate online workshop, which followed a similar structure but without CYP present. Two experienced researchers (DF‐E and GH) co‐facilitated workshops alongside the first author. It was important to have a neuroscientist present (DF‐E) who could answer treatment‐specific questions. Online polls were used to facilitate consensus agreements, and breakout rooms were utilised to facilitate discussions in small groups. The poll results provided parent's views on the acceptable number of treatment sessions, individual session length, frequency, and the logistics of when and where treatment should be carried out (see results in Appendix 2). This helped us rank responses to identify the most preferred choice. Instances of equal responses or topics of no‐agreement were taken to a follow‐up co‐design workshop to seek feedback from additional stakeholders.

Both workshops were audio‐recorded for summary purposes. For those who wanted to take part but could not join the live online workshop, individual meetings were offered. Young people and parents were given vouchers of £15 and £25, respectively (as most parents stayed for both sessions), as a token of appreciation for their time and contributions.

#### 2.2.2 Results

Five CYP with ABI (aged 11–16 years) and 12 parents participated in the co‐design workshops. Both CYP and parents confirmed fatigue as an important area of research worth considering tDCS treatment for. Outcomes identified as important to both parents and young people included feeling less tired, spending more time at school, and engaging more effectively with learning. Following tDCS treatment, CYP specifically wished to be able to play with friends without feeling so tired, to be able to go out more, and to have improved attention, sleep, concentration, and memory. Parents additionally wished to see improvements in their child's sleep quality.

Parents and CYP were not concerned by the technical terminologies associated with electrical stimulation and electrical current, suggesting that older children would be able to comprehend such language, and younger children would not understand what is involved regardless of the language used. Regarding treatment parameters, parents reported a flexible approach, showing equal preference for different treatment frequencies and number of sessions. There was consensus among parents to keep each session length to 20–30 min. Parents preferred for tDCS treatment to be conducted at home, whereas CYP preferred hospital‐based treatment. While YPAG members had suggested morning treatment, parents did not think it was realistic and wanted flexibility to fit the treatment around their usual routines. Both CYP and parents reported feeling happy to try tDCS if it would help manage fatigue. Parents did not anticipate barriers related to equipment use, as many had experience of using similar devices such as transcutaneous electrical nerve stimulation during pregnancy. Parents and CYP were informed about the possible side effects of tDCS, including tingling and burning sensations, and mild headaches. Although parents were unconcerned by these, CYP expressed reservations. CYP also expressed concerns about the uncertainty of treatment effects, placing electrodes on their head, and the uncertainty about how long it would take to see improvements. Parents reported concerns about the logistics of visiting hospital, costs associated with travel, and the impact on work commitments. Poor internet connectivity to complete cognitive assessments and communicate with the research team were also considered to impact their decisions about trying tDCS.

Parents and CYP emphasised the importance of clear communication during the tDCS treatment process. They recommended visual aids such as pictures and video testimonies and recommended the availability of someone with lived experience of using tDCS to learn from. Parents felt it would be beneficial to understand the sensation of tDCS by trying it on themselves first, before attempting to use it on their children. They considered this an essential step in developing confidence and ensuring a successful recruitment process.

When asked about regular engagement in cognitive tasks, as recommended to manage chronic fatigue, and which are typically used alongside tDCS treatment, parents suggested a cognitive activities menu where CYP could select which activities to engage with when receiving tDCS. While this is not advised for the research protocol (tDCS is state‐dependent, and it is necessary to standardise what participants are doing during the stimulation), these optional tasks could be offered following treatment. Parents recommended talking to the family and CYP to explore their interests and encourage continued commitment.

### 2.3 Co‐Design Part 2

#### 2.3.1 Method

The second co‐design workshop was run as a hybrid online/in‐person meeting with professionals and parents of CYP with ABI, alongside the full research team. Here, the aim was to use information gained from the previous CYP and parent workshops to develop a draft research protocol. While the focus was on intervention development and an associated feasibility study, all parameters were chosen within the context of what could be clinically implemented in the longer term. The workshop lasted 2 hours. Participants included clinicians from paediatric neurology, a neuroscientist, a public health practitioner, parents, an academic physiotherapist, a psychologist and qualitative methodologist, a PPIE coordinator, and representatives from the Stroke and Child Brain Injury Trust charities.

Responses from the previous workshops were presented and discussions held to identify barriers to feasibility and mitigation strategies, determine outcome measures, specify inclusion and exclusion criteria, develop a tDCS prototype intervention, and finalise study design, including nested qualitative research. Professionals were asked for feedback to inform the study design related to their area of expertise. All uncertainties surrounding the trial plans were discussed until a consensus was reached. The session was recorded, and a summary was sent to all participants. Findings from this workshop were used to create a prototype tDCS treatment plan and study protocol. Parents were given a £25 voucher to thank them for their time.

#### 2.3.2 Results

Stakeholders felt that CYP should receive tDCS at home, enabling them to go to school. Attending the hospital was considered a significant burden for the child and their family, which would likely increase fatigue. Parents' preferences for delivering treatment at home also had the benefit of being able to increase the frequency of treatment and being more inclusive. Given that the tDCS technology is deliverable at home, it was felt that the study going in that direction would be valuable and cost‐effective. The decision to treat at home led to a discussion about the type of headset to use. It was felt that an inexpensive and easy‐to‐use, portable headset would be more feasible for families over an expensive, sophisticated lab‐based tDCS device. While commercially available headsets are currently only approved to be used by adults, with only small adaptations, they could be used by CYP aged 10 years and above. Parents suggested that we focus on one age group that can express themselves, especially if they are to be treated at home.

Remote support (via telephone and video call) was agreed as beneficial for assisting parents when treating their children at home. For the feasibility study, parents further suggested financially supporting families with travel to the hospital/research centre (for initial assessment), as well as combining any further required assessments (N.B. the study protocol includes MRI scans, and clinical/cognitive assessments) on the same day, to reduce burden. Stakeholders debated including CYP with neurodiversity and agreed to include them to promote greater inclusivity. It was suggested that any changes in treatment plans should be recorded, which could help future studies devise more effective strategies for planning better intervention delivery.

The previous workshop (Co‐design part 1) enabled the research team to identify meaningful efficacy outcomes by mapping out the views of parents and CYP on changes they desired following tDCS treatment (i.e. reduced fatigue and improved sleep quality). These outcomes were discussed in the co‐design part 2 workshop and agreed upon by stakeholders. It was suggested to keep time spent on assessment to a minimum to reduce family burden. The agreed assessments included a computerised cognitive battery from the platform [49, 50, 51, 52]￼, including tasks assessing memory, processing speed, attention, executive function, and emotional processing [53]￼, and validated for remote (at home) use, Pediatric quality of life multidimensional fatigue scale (PedsQL‐MFS, validated for ABI population [54, 55]￼, the Sleep Disturbance Scale for Children (SDCS) [56]￼, and a tDCS side effect questionnaire [57] that parents and CYP could complete at home. While these assessments would capture changes in fatigue, sleep and cognitive functioning, feedback about the study (including experiences of process and suggested areas of change) were agreed to be best captured via post‐trial qualitative interviews.

Factors that might limit recruitment and participation, and mitigating strategies, were discussed in the workshop. In response, the importance of good communication, using simple explanations through various means, was emphasised. Managing parents' expectations from the beginning was also considered critical, as it was suggested that some families may be desperate to try anything that might work. Stakeholders cautioned that initial enthusiasm could wane over time when life gets busy, and adherence to the protocol may be an issue if the children and parents do not experience immediate benefit.

### 2.4 Co‐Refine

#### 2.4.1 Methods

Following the co‐design workshops (parts 1 and 2), the research team drafted a proof‐of‐concept and feasibility trial protocol, along with participant‐facing materials. In the co‐refine stage, discussions centred around tDCS dosing, specifically the length of the stimulation protocol. In earlier stages, we considered a 2‐week protocol length, but this was increased to 4‐weeks. This decision was based on recent evidence suggesting that a minimum of 3 to 4 weeks of daily tDCS is typically required to observe therapeutic changes in chronic conditions, including research in the adult population by the manufacturers of a widely available device for at‐home tDCS delivery [56]. This change was communicated to all parents involved in the previous Co‐design workshops by email.

tDCS has been used in many neurodevelopmental conditions with a positive therapeutic effect and without causing any serious adverse effects. In terms of tDCS intensity, to our knowledge, no formal tDCS dose‐escalation or dose‐finding designs are available for CYP. However, it is estimated that the strength of the electrical field in the target region may be two‐fold higher in CYP as compared to adults, particularly when targeting cortical grey matter regions [30]. Possibly based on this, most of the available studies in paediatric samples typically use lower intensities [58] of 0.75 to 1.5 milliampere (compared to 2 mA being standard in adult studies) [28], but similar individual sessions length (15–30 min), and treatment durations (ranging from 1 to 20 sessions) to adult protocols. Of particular importance, head sizes are more variable (and age dependent) in CYP, and this needs to be considered for the montage. There are also differences in skull size and thickness, cerebrospinal fluid volume, and grey/white matter volumes compared to the mature adult size [59, 60], which may affect conductivity and the electrical field generated with the stimulation [57]. Therefore, a low dose of tDCS (1 to 1.5 mA) in CYP may provide sufficient bioavailability of direct current to the cortex, enhancing neurophysiology without risking structural or functional brain development [28]. Finally, randomisation of active and sham tDCS for the intervention and control groups was discussed, and it was recommended that study participants should be given information about what the sham tDCS entails and why it is needed.

An hour‐long online workshop was arranged with the NIHR‐supported YPAG group (from the co‐define activity). Participants were asked to review and provide feedback on study documents including participant information sheets, consent form and advertisement. Nine participants (10–16 years) and one YPAG coordinator attended this session. All suggested changes were implemented to patient‐facing materials, and the protocol was updated. These documents were then sent to two parents for review (EM and HH).

#### 2.4.2 Results

Parents were happy with an increased protocol duration from 2 weeks to 4 weeks but expressed concerns about an 8 weeks‐protocol, which would be necessary for a crossover trial. Therefore, while we originally considered a crossover trial to mitigate a potentially modest sample size, this was deemed not feasible and we decided on a parallel study instead (active vs control group). However, parents felt very strongly that all participants should receive actual tDCS and thus had concerns about half of the sample being randomised to the sham condition. To address this, we suggested an optional open‐label phase after the main (blind) study period where the control group would be offered 4 weeks of active tDCS with the same protocol used for the active group. This would ensure that all participants had a chance to receive the potentially beneficial tDCS intervention, mitigating ethical concerns around randomisation to placebo conditions in interventional trials [61]. Based on this recommendation, the study design was set to a blind 4‐weeks parallel design with an optional open‐label phase after the study, including 4 weeks of active tDCS for the control group. Parents were happy with this approach.

After reviewing study documents, YPAG members recommended translating the study flyer, using sign language, and adapting the communication style to suit individual children and their families. The consent form presented contained questions relating to pregnancy, smoking, alcohol or recreational drug use (required to establish the safety of the intervention procedures). YPAG members did not feel these questions would be offensive to CYP, but emphasised a need to ensure it would not put CYP participants in an uncomfortable position if these questions were asked while their parents were present. Young people were sceptical whether we would get an honest response from the CYP for these questions, but acknowledged the importance of these factors and how they might influence brain functions.

Parents felt that the intensity of the cognitive activities while receiving tDCS was unclear, and they wondered how this information would be used for analysis. They also recommended that asking parents to engage their children in activities while they received treatment would be a burden. It was therefore decided that participants would not be given a specific cognitive task to complete during the intervention.

The final protocol of FatigueBraIn‐tDCS consists of an initial assessment at the research centre, including training parents' in the safe administration of tDCS at home, as well as completion of a series of outcome questionnaires and a functional MRI scan, 4 weeks of active tDCS intervention at home, and a follow‐up assessment conducted remotely (at an online interview from their homes). Participants will be assigned to a protocol of either anodal (active) or sham (placebo) tDCS stimulation including 4 consecutive weeks of 5 daily sessions (Monday‐Friday, total of 20 sessions, 1 mA dose, and 30 min per session). The control group will be offered an additional 4 weeks of active tDCS after the blinded phase.

The final protocol was peer‐reviewed by an expert clinical psychologist who assesses and treats psychological issues in CYP with ABI. The reviewer's comments and feedback were addressed, and the protocol was amended accordingly. Two parents (EM and HH) also reviewed and provided comments on the protocol, which were addressed. All participant‐facing documents were revised considering comments from parents and members of the YPAG.

## 3 Stakeholder Reflections

### 3.1 Parent 1

As a parent carer of a young person suffering from post‐ABI chronic fatigue, I was heartened to find out about this study as we have had no support for chronic fatigue, and it has had an enormous impact upon my teenage daughter's life. The interactions both myself and my daughter have had with the team have been of a very high level. The study design reflects our discussions and comments, and I can see that the study will be excellently conducted because it has been so thoroughly designed. It incorporates the needs of children and young people with ABI and their families.

I have been involved in lived experience co‐production in the past but have often felt that in the end parent carer and child and young person's voice is not reflected adequately. In this case we have worked with the researcher and the wider team in true coproduction. It has been very rewarding and a pleasure to be part of the team designing this study. I hope this work will make a real difference in the treatment and rehabilitation of children and young people with acquired brain injury in the not‐too‐distant future.

### 3.2 Parent 2

I have worked as a parent voice within the paediatric Type 1 diabetes arena for some time, so I am very aware of how valuable and powerful a parent/patient voice can be in understanding issues, raising awareness and creating solutions, therefore I welcomed the opportunity to get involved with this project.

I found discussing ideas for new research in this area to be timely and crucial given the impact that fatigue is having on my daughter's quality of life. The more interactions that I have with other families through this process and interactions through the Child Brain Injury Trust and the Stroke Organisation, it is evident that most children impacted by ABI are suffering similarly and it appears that this consequence of injury is only just starting to be acknowledged. I was surprised that there is so little research and support to manage the impact of fatigue given the huge impact it has on the child and extended family.

I enjoyed contributing to the project, through discussions and review of papers and methods. It was gratifying to be able to give feedback about how best to produce an effective process and the information material and encouraged to feel that we may finally be heard as to how impactful fatigue is on my child's life and that there may be a therapy that will help in the future.

I felt consultations with parents could be improved by more structure as there were certain parents that monopolised conversations and perhaps diverged from the key remit of the consultation, given the wide range of experiences, time since injury and knowledge of injury impact. However, I appreciated the opportunity to have subsequent 1‐1 interviews to ensure my viewpoint was heard.

## 4 Discussion

Using a structured co‐creation method facilitated positive engagement from stakeholders in developing a complex neurological intervention and protocol for an associated feasibility study. The flexibility of group and individual in‐person, online or hybrid meetings enabled the researchers to learn the different viewpoints of stakeholders. The co‐facilitators were instrumental in managing conflicting views and guiding participants to reach consensus. The co‐design process significantly influenced decision‐making regarding both the treatment format and the research plan. Our initial idea, for example, of delivering tDCS at the hospital was changed due to parents' preference for home treatment. Such an approach also aligns with the NHS 10‐year plan to shift care from acute (hospital) to community settings [62]. This project will serve as a testing ground to determine whether moving treatment from hospital to home in the context of ABI in CYP, enables parents to effectively self‐manage fatigue and to explore the practicalities of clinicians supporting them remotely.

The study direction shifted from researcher‐led to participant‐led in many aspects. For example, parents' preference for home treatment also led to the need for us to source a user‐friendly tDCS device that could be easily and safely used at home, and to the development of protocols to monitor treatment remotely via an online platform. In addition, while we initially considered using a cognitive task during stimulation to enhance the effects of tDCS, this was deemed non‐viable as it would limit accessibility to the intervention in children with greater difficulties, and adherence to the protocol. Importantly, while tDCS is sometimes paired with a task to enhance the effects of the stimulation on the specific cognitive function ‘trained’ by or involved in such a task, individual differences in task performance, demand and difficulty can interfere with the effects of tDCS [63, 64, 65]. In fact, the choice of task can sometimes hinder neuroplasticity and lead to a worsening in performance [66]. When considering an intervention for a cohort with heterogeneous cognitive skills and targeting a non‐specific cognitive function (i.e., fatigue), delivering the stimulation at rest appeared to be the correct choice, not only for feasibility but also in the context of the available scientific evidence. Indeed, most therapeutic uses of tDCS in psychiatric/neurological context use offline protocols where the stimulation is delivered at rest [67, 68].

The final protocol therefore includes tDCS delivered at rest. Some of the researchers' preconceived perceptions were challenged using the co‐creation approach, such as the fear of electric current for participants, which was not an issue for either parents or children. The various stakeholder groups' willingness to modify the project design to extend the treatment length from 2 weeks to 4 weeks, as recommended by scientific evidence for tDCS, will potentially maximise the chances of detecting any beneficial effects while remaining acceptable to families. This shift in the research plan highlights the project's adaptability and responsiveness to stakeholder needs.

Due to the complexity of recruiting people with neurodiversity and intellectual disability, these groups are often excluded from clinical trials [69, 70, 71]. This was the case in the initial research plan (to reduce the heterogeneity of our cohort and due to the complexity of some of the research tools originally considered), but the parents involved in our stakeholder engagement activities strongly felt that including CYP with neurodiversity was vital, and the PPIE groups suggested multiple strategies to include everyone and make the research more accessible.

Finally, creating online and in‐person sessions was time‐consuming and often challenging to accommodate conflicting views of stakeholders, but all views were necessary to manage future implementation challenges. The research team thus became aware of potential logistical issues in advance, through the PPIE meetings, demonstrating the value of stakeholder collaboration in guiding project development and considering implementation issues from the beginning.

Despite the value of this co‐creation work for advancing treatments of fatigue in CYP with ABI, it also has limitations. First, to aid communication, parents were encouraged to support their children in the co‐design workshop with the CYP with ABI. It is possible, however, that their parents' presence may have influenced the responses received from the CYP. In addition, we recognise that our co‐design process represents only a small sample of CYP with ABI. Recruiting a broader range of CYP may have elicited alternative experiences, preferences or needs. While our online meetings increased accessibility, the intervention design reflects only the people we consulted and may not reflect the views of the broader ABI population. For example, we were unable to reach the CYP with ABI aged between 16 and 18 years, who were transferred to adolescent services due to organisational barriers. This has limited our understanding of transition of care related issues, including limited follow‐up access and challenges around young people taking on their own responsibilities within the health and social care context. Similarly, we did not have neuro‐oncology‐related ABI participants for this work; therefore, we are missing their views on the fatigue effects caused by the medical management itself in addition to the impact of the brain injury. Moreover, a few co‐creation sessions were close to school examination times, meaning some CYP were unable to participate; therefore, their views were not reflected in this work. We believe that our approach was appropriate and proportionate to the nature of our study (feasibility trial) and plan to conduct larger‐scale PPIE work to inform our subsequent RCT. According to the NIHR in the UK, PPIE are not considered research or study data collection [72]. Therefore, no ethical approval was required for these PPIE activities. However, some clinicians were reluctant to pass on the PPIE meeting information to their patients due to limited time availability and concerns that we had not sought NHS Health Research Authority approval. This shows a need for PPIE education among some clinicians.

## 5 Conclusion

Our co‐created study protocol prioritised the views and lived experiences of parents and CYP with ABI. This was instrumental in a number of aspects, for instance, in deciding to deliver this intervention at home, including children with neurodiversity, and selecting the age range and session treatment frequency. By involving parent and patient stakeholders early in the research process, we were able to overcome barriers prior to implementation. This co‐created protocol content will be submitted to the NHS ethics committee for approval to assess the feasibility of tDCS for fatigue management in CYP with ABI.

## Author Contributions

**Chandrasekar Rathinam:** conceptualisation, methodology, formal analysis, writing – review and editing, writing – original draft, funding acquisition, investigation, project administration. **Gemma Heath:** conceptualisation, investigation, methodology, writing – review and editing, formal analysis, supervision. **Rajat Gupta:** conceptualisation, writing – review and editing, supervision, formal analysis. **Evangeline Wassmer:** conceptualisation, methodology, writing – review and editing, supervision, formal analysis. **Emily McElroy:** writing – original draft, data curation, formal analysis. **Helen Horn:** writing – original draft, data curation, formal analysis. **Davinia Fernandez‐Espejo:** conceptualisation, investigation, writing – review and editing, project administration, formal analysis, supervision.

## Ethics Statement

According to the National Institute for Health Research (NIHR) in the UK, patient and public involvement and engagement activities (PPIE) are not considered research or study data collection [72]. Therefore, no ethical approval is required for PPIE activities.

## Conflicts of Interest

The authors declare no conflicts of interest.

## Supporting information

Supporting File 1

Supporting File 2

## Data Availability

The participants in this PPIE work did not provide written consent for their data to be shared publicly. Therefore, due to the sensitive nature of this work, supporting data is not available. The data that support the findings of this study are available on request from the corresponding author. The data are not publicly available due to privacy or ethical restrictions.
