# Supplementary material for: Co‐Creation of a Study Protocol to Assess the Effect of Transcranial Direct Current Stimulation in the Management of Fatigue in Children and Young People With Acquired Brain Injury (Fatiguebrain‐tDCS)
Source: Health Expect. 2026 Jun 14;29(3):e70706. doi: 10.1111/hex.70706 (PMC13264680; doi:10.1111/hex.70706)
Supplement: Supplementary file 2 — Supporting File 2 [file HEX-29-e70706-s002.docx]

**Appendix 2**

**Poll results:**

**On a scale from 0 to 10, how important is this work from your perspective?**

Response: 7 - 10

| Total sessions (Response: 5+1*) | Session length (Response: 6+1*) |
| --- | --- |
| 6–10 sessions – 2+1*  16+ sessions – 3 | 20 – 30 min – 3+1*  30 – 45 min - 2  45 – 60 min – 1 |
| Frequency (Response: 6+1*) | Total weeks (Response: 6+1*) |
| 1 – 2 sessions/week – 3  3 – 4 sessions/week – 2+1*  5 – 7 sessions/week – 1+1* | 4 weeks – 5+1*  3 weeks – 1+1* |
| Place (Response: 6+1*) ^ | Who (Response: 6+1*) ^ |
| Home – 4+1*  Hospital – 4 | Hospital people – 5  Parents – 3+1*  Teachers – 1  Combined - 2 |
| When (Response: 6+1*) ^ |  |
| Morning – 5  Evening – 1+1*  Weekdays – 3+1*  Weekend – 2  School holidays – 2 |  |

*  1:1 meeting  ^ Multiple options to choose from for the same questions
